# 2018 Synaptic vesicle 2 receptors as a novel targets for neuroendocrine cancer therapy

**DOI:** 10.1017/cts.2018.125

**Published:** 2018-11-21

**Authors:** Renata Jaskula-Sztul, Jason D. Whitt, Jianfa Ou, Margaret X. Liu, Zviadi Aburjania, Herbert Chen

**Affiliations:** 1 University of Alabama at Birmingham; 2 UAB School of Medicine, Department of Surgery; 3 UAB School of Medicine, Department of Biomedical Engineering

## Abstract

OBJECTIVES/SPECIFIC AIMS: (1) To delineate the function of the heavy-chain receptor binding domain (HCR), a portion of botulinum neurotoxin type A (BoNT/A) and synaptic vesicle 2 (SV2) signaling pathway, which provide a novel multipurpose biologic with potential clinical applications in tumor detection/imaging, inhibition of tumor progression, and reduction of bioactive hormone secretion in metastatic neuroendocrine (NE) cancers. (2) To evaluate the expression pattern of SV2 receptors in NE cancer patient-derived tissues for prediction of patient response for recombinant HCR (rHCR) treatment. (3) To assess the in vivo efficacy and toxicity of rHCR in a NE cancer liver metastasis mouse models and in the NE patient-derived 3D MicroTumor system. (4) To collect preclinical data to design and conduct a clinical trial with NE cancer patients, a major goal toward translating our discoveries into much needed therapies. METHODS/STUDY POPULATION: Recombinant botulinum heavy chain (rHCR) was produced using an IPTG-inducible expression vector in *E. coli* BL21. The rHCR was His-Tag purified and stored in PBS buffer before usage. Cytotoxicity: H727, TT, and MZ cells were plated at a density of 5000 cells/well in 96-well plates and incubated under standard conditions overnight. The next day, cells were treated with 10, 100, or 500 nmol/L of rHCR and incubated for 72 hours. Following incubation, cell viability was assessed by ATP quantification using the CellTiter-Glo (Promega) assay. Fresh NE tumors were dissociated and injected into polydimethylsiloxane bioreactors in a matrigel and collagen suspension for 3D culture experiments. The viability of 3D cultures incubated with various doses of rHCR was assessed by measuring the uptake of the near-infrared dye IR-783 using an IVIS imaging system. Western blot: H727, TT, and MZ cells were seeded in 6-well plates at a density of 3×105 cells/well for 24 hours followed by treatment with 100 nmol/L for 72 hours. Total cellular proteins were isolated and analyzed to assess the level of SV2A expression and the effect of rHCR on the expression levels of NET marker proteins. Immunohistochemistry: Deparaffinized tissue culture slides were incubated with SV2A primary antibody in 1% BSA and incubated overnight at 4°C. Slides were rinsed twice with TBS containing 0.025% Triton, followed by 0.3% H_2_O_2_ for 15 minutes. Slides were then incubated with HRP-conjugated secondary antibody for 1 hour at room temperature. Detection of protein-protein interaction: Precleared cell lysate was incubated with glutathione-agarose beads in the presence of 10 μg of GST-tagged rHCR for 2 h at 4°C with end-over end mixing. Samples were then centrifuged at 10,000 g for 2 minutes and the supernatant was analyzed by SDS-PAGE. Preclinical models: To allow rHCR testing on NET patient derived cells in a very novel 3D surrogates, sterile collected fresh NET tissues will be obtained from the UAB Tissue Procurement, dissociated into a single cell suspension and injected into a polydimethylsiloxane bioreactors containing extracellular matrix composed of bovine collagen and matrigel. Such 3D cell culture will be maintained in bioreactors with constant supply of media through the microchannels and treated with rHCR at concentrations ranging from 10 nM to 1 mM. Following histologic confirmation of growth and morphology of NET patient-derived 3D surrogates we will test the anticancer activity of rHCR in this system using the standard cytotoxicity assays as well as we will validate the NET hormone expression using immunohistochemistry assay. To create an animal model of NE cancer progression, we will perform intrasplenic injection of NET cell lines. In approximately 4 weeks, the animals should develop NE liver metastases based upon our previous experience. rHCR-iFPs accumulation in the tumor mass: rHCR-iFPs will be injected to the tumor bearing mice after 4 weeks of cells implantation in 1 week interval for total of 4 treatments at the concentrations of 0.125, 1.25, and 12.5 mg/kg. RESULTS/ANTICIPATED RESULTS: Based on the preliminary data, we expect to detect rHCR-iFPs in NE cancer xenografts and in patient derived 3D explants. Our preliminary data revealed that treating NETcells with rHCR significantly reduced NE peptide expression in 3 days. Thus, we expect to see the decrease of NE tumor markers even if the fluorescent detection method is not sensitive enough to monitor the signal. The reduction of the NET markers can be used as an indicator of the rHCR-iFPs uptake by the tumor mass. If HCR exhibit high binding affinity to SV2 receptors in NET models but moderate anticancer efficacy, we plan to use rHCR peptide to conjugate with the nanocarrier for targeted drug delivery. In this case rHCR peptide can be used as a ligand that specifically binds to NE cancer cells and delivers anticancer drug. In 3D NET patient derived explants we expect significant reduction of NET markers and hormones (serotonin and calcitonin) in NE cancer cells upon long-term rHCR-iFPs treatment. In addition, we will perform multiplex protein quantification assay using Luminex to assess the various hormones, cytokines, and growth factors to be repurposed into a diagnostic and detection reagent, or a drug delivery ligand for targeted therapies. DISCUSSION/SIGNIFICANCE OF IMPACT: NE cancers are highly metastatic: NE cancers such as carcinoid, islet cell tumors, and medullary thyroid cancer frequently metastasize to the liver. They are the second most prevalent GI malignancy. Ninety percent of patients with pancreatic carcinoid tumors and 50% of patients with islet cell tumors develop isolated hepatic metastases. Patients with untreated, isolated NE liver metastases have <30% 5-year survival. Thus, there is a critical need for new therapies for NE cancers. Surgery is the only curative therapy available for patients with NE cancers but most cannot be cured: surgical removal is the most effective treatment for NE cancers; however, a very high percentage of patients present with metastatic disease. While surgical resection can be potentially curative, many patients are not candidates for operative intervention due to widespread metastases or the degree of hepatic involvement by the NE cancers. Moreover, other forms of therapy including chemoembolization, radioembolization, cryoablation, and chemotherapy have had limited efficacy. We hope that our in vitro data on rHCR toxicity and specificity to NET, validated in the pre-clinical models will allow the first in-human application of this technology in clinical studies.

